# Linkage analysis of GAW14 simulated data: comparison of multimarker, multipoint, and conditional approaches

**DOI:** 10.1186/1471-2156-6-S1-S40

**Published:** 2005-12-30

**Authors:** Mathew J Barber, Eleanor Wheeler, Heather J Cordell

**Affiliations:** 1 Department of Medical Genetics, University of Cambridge, Cambridge Institute for Medical Research (CIMR), Wellcome Trust/MRC Building, Addenbrooke's Hospital, Cambridge, CB2 2XY, UK Department of Medical Genetics, University of Cambridge, UK

## Abstract

The purposes of this study were 1) to examine the performance of a new multimarker regression approach for model-free linkage analysis in comparison to a conventional multipoint approach, and 2) to determine the whether a conditioning strategy would improve the performance of the conventional multipoint method when applied to data from two interacting loci. Linkage analysis of the Kofendrerd Personality Disorder phenotype to chromosomes 1 and 3 was performed in three populations for all 100 replicates of the Genetic Analysis Workshop 14 simulated data. Three approaches were used: a conventional multipoint analysis using the *Zlr *statistic as calculated in the program ALLEGRO; a conditioning approach in which the per-family contribution on one chromosome was weighted according to evidence for linkage on the other chromosome; and a novel multimarker regression approach. The multipoint and multimarker approaches were generally successful in localizing known susceptibility loci on chromosomes 1 and 3, and were found to give broadly similar results. No advantage was found with the per-family conditioning approach. The effect on power and type I error of different choices of weighting scheme (to account for different numbers of affected siblings) in the multimarker approach was examined.

## Methods

Linkage analysis of the Kofendrerd Personality Disorder (KPD) phenotype to chromosomes 1 and 3 was performed in the Danacaa, Karangar, and Aipoto populations, with knowledge of the "answers". An important aim of our investigation was to compare the results from an affected sib-pair (ASP) multimarker approach with those from a conventional multipoint approach, and these populations were chosen because of their ascertainment via nuclear families rather than via multi-generational pedigrees. Multipoint linkage analysis was performed using the allele-sharing *Zlr *statistic [1] as calculated in the program ALLEGRO [2] under an exponential model. Since it was known from the "answers" that the disease loci on chromosomes 1 and 3 interact in an epistatic manner, we also performed a weighted conditional analysis in which the per-family contribution to the *Zlr *on one chromosome was weighted according to evidence for linkage on the other chromosome, as previously suggested [3].

The results from the multipoint approach were compared with those from a multimarker regression approach that models the observed identity-by-descent (IBD) states for ASPs at a series of genetic markers in terms of the IBD state at a presumed disease locus in the region. The expected IBD state at the disease locus, and hence the expected IBD state at the marker loci, are considered parameters to be estimated in the regression procedure. For a given marker and parent type (mother or father), the expected IBD state can be written as *p*_*M *_= *x*_*1 *_+ *p*_*D*_*x*_*2*_, where *p*_*M *_and *p*_*D *_correspond to the probability of sharing an allele IBD at the marker and disease locus, respectively, and the *x *variables correspond to conditional probabilities of marker IBD state given disease locus IBD state: *x*_*1 *_= *P(M|d) *and *x*_*2 *_= *P(M|D) - P(M|d)*. Here *M *and *m *denote the events that the observed marker IBD state is 1 and 0, and *D *and *d *the events that the disease IBD state is 1 and 0, respectively. These may be written *P(M|D) = θ^2^+(1 - θ)^2 ^*and *P(M|d) = 1 - P(m|d) = 1 - P(M|D)*. Thus, the expected IBD states at each of the markers are modelled in terms of *p*_*D*_, the expected IBD state at the disease locus (which will be estimated as a regression coefficient), and *x *variables that are functions of the recombination fractions *θ *between the markers and disease locus. The IBD states for mothers and fathers are modelled separately (assuming independence), which allows the possibility of using different values of *θ *for the two types of parent, i.e., incorporating sex-specific recombination fractions if desired.

**Figure 1:**
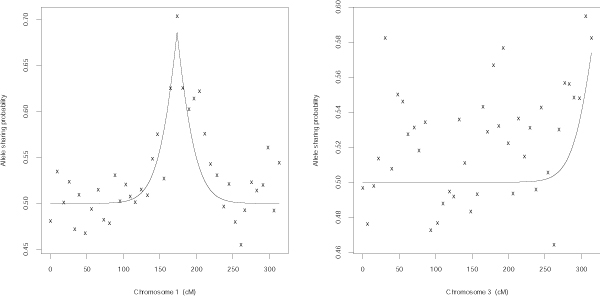
Example of multimarker approach for Danacaa population, replicate 100. Results are shown for the fitted regression line that maximizes the test of *p*_*D *_= 0.5 across each chromosome.

The model specifying the expected IBD states is fitted to the observed marker IBD states via a generalized estimating equation (GEE) approach. Because the IBD state is considered for each parent separately, the observed IBD events are Bernoulli random variables with known functional relationship between the mean and variance, and correlation between IBD states (at different markers for a given parent type) that depends on *p*_*D *_and the known recombination fractions between the markers. The data may be analyzed via standard GEE software that allows specification of the correlation structure (specified under the null hypothesis that *p*_*D *_= 0.5). At a given putative disease locus location, this procedure provides an estimate  of *p*_*D *_together with its estimated standard error SE () that may be used to produce a *z*-score ( - 0.5)/SE () that is normally distributed under the null hypothesis that *p*_*D *_= 0.5. The whole procedure is repeated with the disease locus allowed to take a variety of putative positions along the marker map, and the position where the *z*-score is most significant is taken as the estimate of the disease locus location. An example of the fitted regression line, using the disease locus location that gives maximal evidence against the null hypothesis, is shown in Figure 1 for chromosomes 1 and 3 of the Danacaa data, replicate 100.

The multimarker approach is both conceptually and analytically very similar to a previously proposed GEE approach [4]. The multimarker approach differs from the previously proposed method mainly with regard to the test statistic, which is calculated at a variety of increments (putative positions of the disease locus) across the region, in an approach akin to standard multipoint analysis. The multimarker approach also differs from the previously proposed approach by considering the contribution of each parent separately, which could potentially allow the use of different marker maps in males and females (although sex-specific maps were not provided for these data). From Figure 1, it is clear that the greatest contribution to the test statistic at a given disease locus location will come from the observed IBD states at the two flanking markers. The speed of the multimarker procedure can therefore be considerably increased by using data only from the two flanking markers, in an interval mapping type approach, when testing a putative disease location. For each parent, we used data from the two flanking markers (when informative) or the closest informative flanking markers otherwise. In practice, this appeared to make very little difference to the multimarker results (data not shown) and so results presented here will all assume the flanking marker approximation.

An issue not investigated in the previously proposed approach [4] was the choice of different possible weighting schemes for ASPs derived from sibships with more than two affected individuals. Several different weighting schemes have been proposed to adjust for the non-independence of such affected pairs, but the optimal scheme will depend both on the analysis method used and on whether the goal is merely to maintain type I error or also increase power [5]. With regard to power, the optimal weighting scheme may depend on the unknown underlying genetic model [5]. We investigated the performance of four different weighting schemes for the multimarker approach. The schemes investigated were 1) the Hodge scheme [6], in which the contribution of each ASP from a sibship with *a *affected individuals is scaled by a factor of (4/3)(2*a*-3+0.5^*a*-1^)/[a(a-1)]; 2) the Suarez and Hodge scheme [7], in which each ASP's contribution is scaled by a factor of 2/*a*; 3) the scheme used by Liang et al. [4], in which each family contributes equally, achieved by scaling the contribution of each pair by a factor of 2/[*a*(*a*-1)]; 4) a scheme where each pair contributes equally regardless of the number of affected sibs in the family. These weighting schemes were incorporated into the multimarker analysis via use of importance weights in the statistical analysis package STATA.

## Results

**Figure 2:**
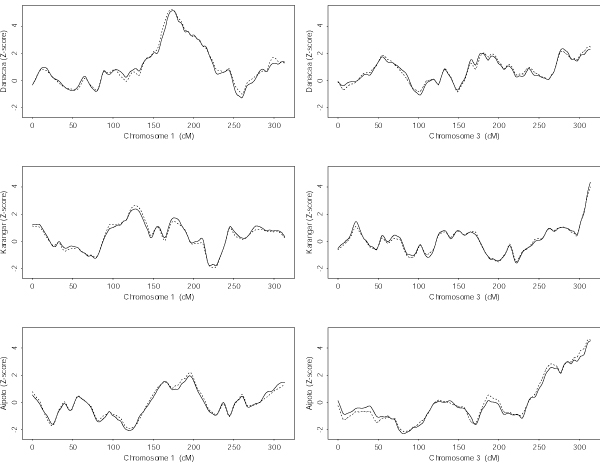
Comparison of multipoint results (shown with solid lines) and multimarker results (shown with dashed lines) for replicate 100.

Figure 2 shows the results from the multipoint and multimarker (with Hodge weights) approaches applied to a single replicate, replicate 100. Results are very similar for both methods. The Danacaa study appears to provide good evidence for the disease locus on chromosome 1, but the results on chromosome 3 are less convincing. The Karangar and Aipotu studies show little evidence of linkage on chromosome 1 but provide good evidence of linkage for the disease locus on chromosome 3. The results for the multipoint analysis of all 100 replicates are shown in Table 1. The average maximum *Zlr *on each chromosome is slightly higher than the average *Zlr *at the true disease locus location, as expected, owing to the upward bias incurred by choosing the maximum on a chromosome. The Danacaa study generally provides good evidence for the disease loci on chromosomes 1 (mean *Zlr *= 4.52, *p *= 3 ×10^-6^) and 3 (mean *Zlr *= 3.92, *p *= 4 ×10^-5^). The Karangar study provides reasonable evidence for the disease locus on chromosome 3 (mean *Zlr *= 2.80, *p *= 0.002) but little evidence on chromosome 1 (mean *Zlr *= 1.32, *p *= 0.09), while the Aipotu study provides good evidence for the disease locus on chromosome 3 (mean *Zlr *= 3.20, *p *= 0.0007) and some evidence for the disease locus on chromosome 1 (mean *Zlr *= 2.08, *p *= 0.02). The *Zlr *scores from the conditional weighted analyses are lower than those from the unweighted analysis, indicating no advantage from using conditioning weights.

**Figure 3:**
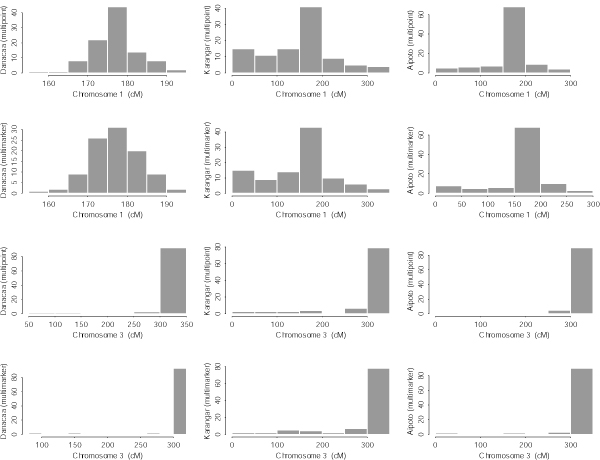
Histograms showing location of maximum over 100 replicates for multipoint and multimarker methods.

The *z*-score results from the multimarker approach are given in Table 2, and are found to be broadly comparable with the multipoint results, particularly when using the Hodge or Suarez and Hodge weighting schemes. Type I error is acceptable for all four weighting schemes, as shown in Table 2 by the analysis of chromosome 4, on which no disease locus exists. The mean *z*-score on chromosome 4 is close to 0 with variance close to 1 and approximate normality (and therefore correct type I error, data not shown) for all four weighting schemes. The positions of the maximum *Zlr *from the multipoint approach and the maximum *z*-score from the multimarker approach are shown in Figure 3. Localization of the disease loci (at true positions approximately 173 cM on chromosome 1 and 314 cM on chromosome 3) is generally good for both methods, although there is some suggestion that the localization on chromosome 1 in the Danacaa population is slightly more precise under the multipoint approach.

## Discussion

Overall, the multimarker and multipoint approaches appear to provide quite similar results, particularly when using the Hodge or Suarez and Hodge weighting schemes. Slightly greater power for the multimarker approach is obtained using the 'Equal pairs' weighting scheme, which is consistent with the results of Sham et al. [5]. The generally stronger results from the Danacaa study in comparison to the Karangar and Aipotu studies are perhaps not surprising, given that the ascertainment of the Danacaa families is via phenotype 1, which is influenced solely by the disease loci on chromosomes 1 and 3.

The *Zlr *scores from the conditional weighted analyses are lower than those from the unweighted analysis, indicating no improvement in power from using conditioning weights, and no power to detect an interaction. The exact form of the proposed interaction is not specified in the "answers" and could potentially correspond to a number of different underlying scenarios [8]. Only those scenarios that result in departure from a multiplicative penetrance model might in fact be expected to be detectable using the approach described here.

## Conclusion

The multipoint and multimarker approaches were generally successful in localizing known susceptibility loci on chromosomes 1 and 3, and were found to give broadly similar results. No advantage was found with a per-family conditioning approach. For the multimarker approach, greatest power and acceptable type I error was seen with the 'Equal pairs' weighting scheme.

## Abbreviations

ASP: Affected sib pair

GEE: Generalized estimating equation

KPD: Kofendrerd Personality Disorder

IBD: Identity by descent

## Authors' contributions

MJB developed and applied the multimarker regression approach. EW applied the per-family weighted multipoint approach and generated the figures. HJC directed the project and drafted the final manuscript.

## Figures and Tables

**Table 1:** Average *Zlr z*-score (over 100 replicates) using multipoint and weighted conditional analysis

		Danacaa				Karangar				Aipotu			
		
		Maximum *z*		*z *at true location		Maximum *z*		*z *at true location		Maximum *z*		*z *at true location	
		
Chr	Conditioning weights^A^	Mean	SD	Mean	SD	Mean	SD	Mean	SD	Mean	SD	Mean	SD
1	Unweighted	4.83	0.89	4.52	0.96	2.35	0.72	1.32	1.14	2.70	0.74	2.08	0.97
	Weights 0–1	-^b^	-	3.30	1.07	-	-	0.73	1.12	-	-	1.49	0.96
	Weights 1–0	-	-	2.09	1.18	-	-	0.80	1.06	-	-	1.18	0.95
	Weights NPL	-	-	2.95	1.00	-	-	0.67	1.09	-	-	1.30	1.05
	Max weights	-	-	3.55	0.90	-	-	1.39	0.89	-	-	1.99	0.74
3	Unweighted	3.99	1.05	3.92	1.11	3.06	0.81	2.80	1.03	3.36	0.97	3.20	1.06
	Weights 0–1	-	-	3.13	1.10	-	-	1.91	1.07	-	-	2.31	1.04
	Weights 1–0	-	-	2.19	1.12	-	-	2.02	0.94	-	-	2.09	1.19
	Weights NPL	-	-	2.74	1.19	-	-	1.51	1.06	-	-	1.94	1.01
	Max weights	-	-	3.43	1.02	-	-	2.56	0.79	-	-	2.91	0.93

^a^Weights 0–1, 1–0 and NPL are described by Cox et al. [3], where Weights NPL is called weight_PROP_. Max weights corresponds to the maximum *Zlr *under the 0–1, 1–0, and NPL weighting schemes.

^b^-, results not calculated.

**Table 2:** Average *z*-score (over 100 replicates) using multimarker analysis with various sibship weighting schemes

		Danacaa				Karangar				Aipotu			
		
		Maximum *z*		*z *at true location		Maximum *z*		*z *at true location		Maximum *z*		*z *at true location	
		
Chr	Weighting Scheme	Mean	SD	Mean	SD	Mean	SD	Mean	SD	Mean	SD	Mean	SD
1	Hodge	4.99	0.83	4.67	0.89	2.33	0.75	1.35	1.16	2.74	0.78	2.15	1.02
	Suarez & Hodge	4.93	0.82	4.62	0.88	2.32	0.74	1.32	1.15	2.72	0.77	2.11	1.01
	Equal families	4.42	0.74	4.1	0.83	2.24	0.65	1.12	1.08	2.54	0.72	1.82	1.00
	Equal pairs	5.16	0.92	4.85	0.96	2.41	0.85	1.46	1.24	2.83	0.84	2.24	1.06
3	Hodge	4.04	1.04	3.95	1.11	3.03	0.76	2.78	0.99	3.39	0.97	3.23	1.05
	Suarez & Hodge	4.02	1.03	3.94	1.10	3.02	0.76	2.76	0.99	3.36	0.97	3.2	1.05
	Equal families	3.77	0.96	3.68	1.04	2.86	0.73	2.56	0.98	3.08	0.94	2.86	1.04
	Equal pairs	4.03	1.07	3.94	1.16	3.05	0.79	2.78	1.01	3.46	0.98	3.28	1.09
4	Hodge	-^a^	-	0.01	0.98	-	-	0.03	1.04	-	-	0.05	1.01
	Suarez & Hodge	-	-	0.00	0.98	-	-	0.03	1.03	-	-	0.05	1.01
	Equal families	-	-	-0.02	1.00	-	-	0.04	1.03	-	-	0.07	1.02
	Equal pairs	-	-	0.03	0.98	-	-	0.02	1.04	-	-	0.03	1.01

^a^-, results not calculated.
